# Using body mapping to explore perceptions of resilience with 7–12‐year‐old Muslim children in East London: A qualitative study

**DOI:** 10.1002/jcv2.70088

**Published:** 2026-04-16

**Authors:** Aisling Murray, Imogen I. Hensler, Eleanor Keiller, Faiza Durrani, Patrisiya Ali Taleb, Maria Grazia Turri, Jennifer Y. F. Lau

**Affiliations:** ^1^ Youth Resilience Unit Centre for Psychiatry and Mental Health Wolfson Institute of Population Health Queen Mary University of London London UK; ^2^ Department of Experimental Psychology University of Oxford Oxford UK; ^3^ Centre for Preventive Neurology Wolfson Institute of Population Health Queen Mary University of London London UK; ^4^ Department of Psychology Brunel University London London UK; ^5^ Centre for Psychiatry and Mental Health Wolfson Institute of Population Health Queen Mary University of London London UK

**Keywords:** child development, qualitative methods, resilience, social factors

## Abstract

**Background:**

Understanding resilience in pre‐adolescence is important for informing interventions to promote good mental health. Middle childhood is a critical developmental phase, characterised by significant emotional and behavioural development. However, there is limited research on children's perceptions and diverse experiences of resilience which could inform interventions. Qualitative methods can enable meaningful engagement of children and provide rich insights into perceptions of resilience. This study involved Muslim children in East London, a population disproportionately affected by deprivation and racial and cultural discrimination yet underrepresented in resilience research. This study aimed to explore (1) children's perceptions, meanings and experiences of resilience, and (2) the factors and resources that constrain and contribute to resilience in Black and South Asian Muslim children aged 7–12 living in East London.

**Methods:**

Data were collected through a one‐day workshop at a community centre, during which one of the activities was body mapping with children (*n* = 12). Body mapping, a visual arts‐based research method, was used to explore children's subjective and embodied experiences of resilience. Here, we report on the findings from body mapping with children. Themes were developed using systematic visuo‐textual analysis and reflexive thematic analysis.

**Results:**

Findings are grouped into (1) Conceptualising resilience and (2) Personal and social resources for resilience. Children viewed resilience as personal strength, and related it to nature, sports and physical health. External support from family, friends, teachers and role models was also highlighted as important for resilience.

**Conclusion:**

This study provides insights into how children conceptualise resilience and the resources they view as important for promoting it. The findings contribute to understanding resilience in middle childhood and highlight the value of resource‐oriented approaches for resilience‐supporting interventions. Body mapping emerged as an effective method for engaging children creatively and non‐verbally on this topic.

## Key Points

**What's known?**

- Understanding resilience in pre‐adolescents, particularly among underrepresented populations, is vital for developing effective mental health interventions, yet limited research exists on children's diverse experiences of resilience.

**What's new?**

- This study used body mapping, a novel approach to explore children's creative and embodied perspectives on resilience.
- Children conceptualised resilience as personal strength, relating it to nature, sports and physical health, and external support from family, friends, teachers and role models.

**What's relevant?**

- The study supports calls for resource‐oriented and socioecological approaches for mental health interventions to promote resilience in children.
- Future research should explore resilience from children's perspectives to inform specific factors and resources which can be targeted through resilience‐supporting interventions.

## Graphical abstract

This study explored resilience in Black and South Asian Muslim children aged 7–12 in East London, an underrepresented group affected by deprivation and discrimination. Using body mapping, children depicted resilience as personal strength and described the importance of support systems. Our findings contribute to understanding resilience in middle childhood and highlight the value of resource‐oriented approaches for resilience‐supporting interventions.

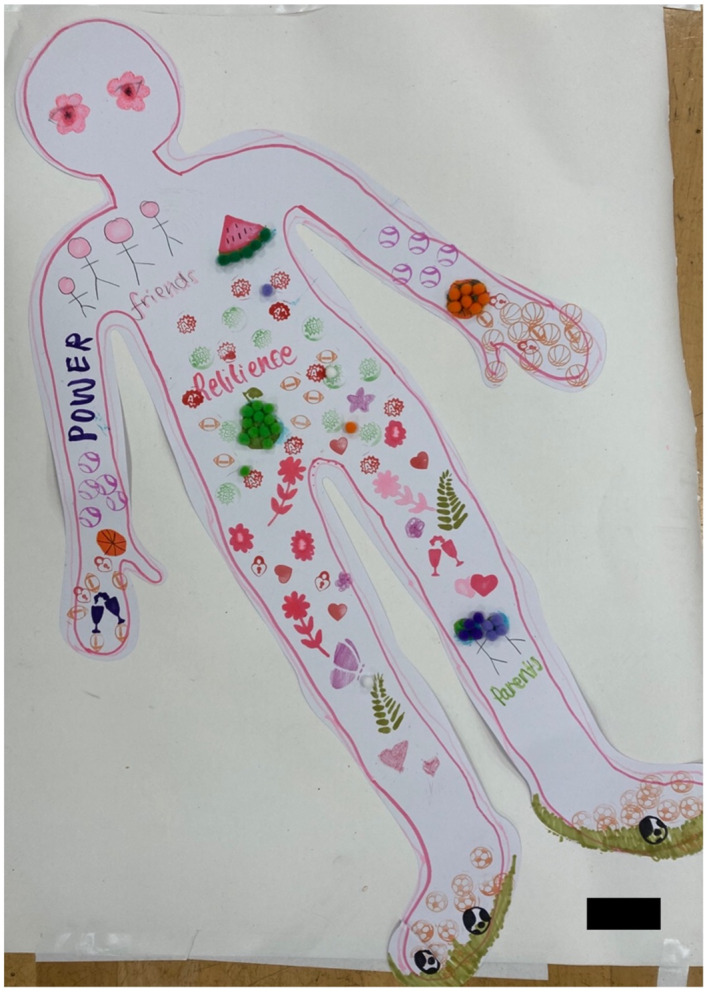

## INTRODUCTION

Rates of mental health disorders in children and young people (CYP) have risen in recent years (Cybulski et al., 2021; Houtrow et al., 2014; Piao et al., 2022), particularly since the COVID‐19 pandemic (Kauhanen et al., 2023; Racine et al., 2021). Many children are considered ‘at‐risk’ of failing to thrive due to chronic stressors within the family and wider environment (Poole et al., 2017; Rak & Patterson, 1996; Wright et al., 2019). Certain groups are more at‐risk, including CYP from minoritised backgrounds who are more likely to experience poverty, low socioeconomic status and discrimination (Ingleby, 2012; Silva et al., 2016).

In the UK, Muslims, the country's second‐largest faith group (Muslim Council of Britain, 2022), are predominantly from Asian/Asian British (65.9%) and Black, African, Caribbean or Black British (10.8%) backgrounds (Muslim Council of Britain, 2024, 2025). Many Muslims live in areas of high relative deprivation (Muslim Council of Britain, 2022) and child poverty (Joseph Rowntree Foundation, 2022), and face increasing racial, religious and cultural discrimination (Muslim Council of Britain, 2025). These social determinants contribute to poorer mental health outcomes (Jaspal & Lopes, 2021; Samari et al., 2018). A recent survey reported increased anxiety among Muslim adults and their children in the UK following the Islamophobic riots after the stabbings in Southport in July 2024 (Gohir, 2024).

These experiences impact CYP from Muslim families (Muslim Council of Britain, 2025). A survey of young Muslims (18–30 years) found high rates of anxiety (53.8%), depression (49.4%) and stress (48.6%) coupled with shame, stigma and mental health illiteracy as barriers to help‐seeking (Bunglawala et al., 2021). Additionally, Gohir (2024) reported cases of children witnessing verbal and physical assaults and experiencing bullying following the 2024 riots. With the youngest age profile of all faith groups in the UK, with 48% under the age of 25 in 2011, including one‐third aged 15 and under (The Muslim Council of Britain, 2015), early interventions for Muslim CYP are critical to reduce the cumulative effects of disadvantage across the life‐course (Citizens Commission on Islam, 2017).

Evidence suggests that greater resilience is associated with fewer mental health difficulties in CYP (Mesman et al., 2021). Middle childhood, a period of significant emotional development and increased risk of onset of mental disorders (Costello et al., 2003; Kessler et al., 2005), remains underexplored in resilience research (Gartland et al., 2021; Windle et al., 2011). Promoting resilience in middle childhood may help prevent the development of mental health difficulties by strengthening CYP's internal resources and capacity to access resources in their social, built and natural environments to cope with stress and adversity (Ungar & Theron, 2020). Resilience is a dynamic process shaped by psychological, biological, social, economic, political and environmental factors (Wathen et al., 2012). This socioecological perspective emphasising how individual processes or outcomes are embedded within wider social contexts (Vaughn & DeJonckheere, 2021). Given the impact of structural inequalities, resilience may buffer the impact of adversity for CYP from minoritised backgrounds, supporting them to regain, maintain or improve their mental wellbeing (Ungar & Theron, 2020). Critically, protective resources differ across populations, meaning that resilience‐promoting interventions must be tailored to CYP's lived realities (Poole et al., 2017; Theron, 2016; Wright et al., 2019) and target multiple systems—for example, parents, families, schools, and communities—rather than relying on a ‘one‐size‐fits‐all’ model (Shean, 2015; Theron, 2016; Ungar, 2011).

To contribute to the evidence base on children's contextual experiences, this study explored resilience among Black and South Asian Muslim children in East London, which one of the largest Muslim populations in the UK (Muslim Council of Britain, 2022). Few studies have explored resilience in this age group from children's own perspectives, particularly those from underrepresented groups (Gartland et al., 2019). Qualitative research with minoritised groups prioritises local knowledge, challenging dominant Western discourse which may be less relevant, or even harmful, to children in diverse contexts (Ungar, 2005; Ungar et al., 2007). To centre children's perspectives, we selected a qualitative, arts‐based approach to capture diverse and nuanced experiences (Shean, 2015). In contrast to traditional verbal qualitative methods (Bergström et al., 2019), visual arts‐based methods, such as body mapping, use symbolism, metaphor, and artistic processes to go ‘beyond logical verbal language to come to an understanding of voice’ (Caldairou‐Bessette et al., 2020). Defined as ‘creating body maps using drawing, painting or other art‐based techniques to visually represent aspects of people's lives, their bodies and the world they live in’ (Gastaldo et al., 2018), body mapping involves tracing the body to produce a life‐sized outline then filled with words, colours and symbols (de Jäger et al., 2016). This method allows for abstract, non‐linear and embodied narratives to be conveyed non‐verbally (Collings & Smith, 2021).

We used body mapping with Black and South Asian Muslim children to address two research questions: (1) what are the perceptions, meanings and experiences of resilience, and (2) what are the factors and resources that contribute to resilience in children? These data will contribute to the evidence base on child resilience to inform preventative programmes and policies (Gartland et al., 2021). To ensure the transparency of our research (Frohwirth et al., 2023), we pre‐published our study protocol (Murray et al., 2024).

## METHODS

### Study design

#### Theoretical framework

This study was informed by embodied inquiry, a research approach centred on embodied lived experiences (Leigh & Brown, 2021). As it is not a theory or single method in itself, embodied inquiry is rooted in phenomenology, multimodal communication and hermeneutics (Leigh & Brown, 2021). It draws from phenomenology the idea that the essence of human experiences and phenomena are embodied (Connelly, 2010; Merleau‐Ponty & Bannan, 1956), asserting that humans are relational and contextual beings who interpret the world and their experiences personally (Leigh & Brown, 2021). This aligns body mapping, which enables exploration of how participants experience their lives within their bodies and environments (Gastaldo et al., 2018).

Unlike traditional phenomenological studies which rely on verbal qualitative methods, embodied inquiry draws on multimodal methods (Leigh & Brown, 2021). Body mapping suits this approach as it involves physical (body tracing), visual (depictions on the map), verbal (describing the body map) and relational (interacting with participants and researchers) modes of expression (Dew et al., 2018). This multimodality supports ‘personal reflection, sensory immersion and relational exchange’ (Collings et al., 2021, p. 880), enabling participants to explore difficult‐to‐articulate feelings in ‘ways that are safe and supportive’ (Orchard, 2017, p. 2) and extend beyond verbal communication.

### Study procedures and participants

To ensure cultural relevance, we consulted a group of parents from an Islamic primary school in East London to review participant‐facing documents and provide feedback on the relevance and appropriateness of our research questions and methods. Additionally, the body mapping process, adapted from Malchiodi (2020), Gastaldo et al. (2012) and McCorquodale and DeLuca (2020), was checked for age appropriateness by EK, a dramatherapist with experience working with children, and community acceptability by FD and PT, who are both Muslim.

We conducted this study at a community centre connected to a mosque in Tower Hamlets, which offers community services primarily for women. We used purposive sampling for recruitment. Families were eligible if they were Black or South Asian Muslims living in East London with children aged 8–12, proficient in English, able to provide written informed consent, including to being audio‐recorded. Up to two children per family could participate to ensure a wide sample of families. Recruitment initially involved physical and digital posters advertised through the community centre. However, it ultimately relied on face‐to‐face recruitment by FD at her local mosque, making mothers with which FD had existing rapport the entry point for recruitment. Interested mothers completed an online REDCap form to confirm eligibility. As per our community consultation, to reduce power dynamics and encourage openness, only mothers and children were invited to the workshop. Following the workshop, AM contacted participating mothers to try and recruit their partners for a focus group.

Of 12 families who expressed interest, two families dropped out due to illness (*n* = 1) and no longer wishing to take part (*n* = 1) and one family did not meet the inclusion criterion of proficiency in English. Of the four fathers who originally expressed interested, two dropped out due to other commitments. The final sample included nine mothers, two fathers and 12 children. Owing to the different data types gathered from children and parents, and to enable a rich and in‐depth exploration of the findings, this paper only reports findings from body mapping with children. The findings from parents will be described in detail elsewhere (Murray, Hensler, et al., 2025).

#### Data collection

We conducted a 3.5 h workshop with mothers and children, and a 90‐min interview with fathers. The workshop began with mothers completing a demographic survey comprising questions related to them and their child on education, employment, family structure and home life. Next, the whole group took part in participatory warm‐up activities before mothers and children separated into groups. Mothers took part in focus groups (1.5 h), one group facilitated by FD and one by PT, whilst all 12 children completed body mapping (1.5 h), facilitated by EK with support from AM and JYFL. Following the workshop, AM and PT facilitated a 90‐min interview with two fathers at a university location.

Body mapping involved using A1 paper rolls (841 mm × 10m roll) to either trace participants' bodies, with assistance from a researcher (EK or AM), or use a pre‐made outline. All but one participant chose to use a pre‐made outline. Craft materials were provided, including stamps, pens, coloured paper, post‐it notes, stencils, pom‐poms and pipe cleaners. Participants were guided through the process with prompts related to embodied experiences of resilience (Murray et al., 2024). Participants then either described their body maps to the group of children or individually to one of the researchers (AM or EK). They were asked to reflect on their body maps and the process of body mapping (Murray et al., 2024), with other participants also invited to ask questions. Due to time constraints and preference, three participants provided verbal or written descriptions of their body maps after the workshop, sent to AM by their mothers. Children could choose whether to keep their body maps after the workshop.

All sessions followed a semi‐structured topic guide (Murray et al., 2024), were audio‐recorded and conducted in English. Each family was offered an £80 voucher for their participation in the workshop and fathers were offered a £30 voucher for their participation in the interview.

#### Sample size

Whilst sample sizes in body mapping research vary (from 3 to approx. 50), our sample of 12 is in line with the recommended 6–12 range to capture rich data on a range of experiences (Macken et al., 2021). Given our embodied inquiry and reflexive thematic analysis approach, data saturation was not sought in this study. Given the open, fluid and organic process of reflexive thematic analysis, we focused on achieving depth and richness through sustained engagement with the data and iterative, reflexive interpretation (Braun & Clarke, 2021). Our sample size reflects ‘a mix of interpretative, situated and pragmatic judgement’ (Braun & Clarke, 2021, p. 211), whereby it reflects our theoretical and analytical positions as well as the practicalities of accessing our target population and working within the schedule of the community centre.

### Analysis

We analysed the body mapping data using systematic visuo‐textual analysis (Brown & Collins, 2021), which views visual and textual data as equally important. This involves weaving between two levels—(1) noticing and describing and (2) conceptualising—and three elements—(1) visual only, (2) transcripts only and (3) visuotextual combined (see Brown & Collins, 2021). As this framework enables flexible application of an analysis approach, we used reflexive thematic analysis given its iterative nature and focus on researchers' active role in interpretation of the data, the latter suiting our dataset of majority visual data (Braun & Clarke, 2019, 2023; Braun et al., 2022).

The first author (AM), a social scientist with body mapping research experience, led the analysis, with support from IH, also a social scientist. The involvement of two researchers enabled sense checking and reflexive discussion throughout the analysis (Byrne, 2022). As per reflexive thematic analysis, analysis began with AM and IH looking over the visual and verbal datasets to gain familiarity. We then independently inductively coded the data at level one, in line with the preliminary coding stage of reflexive thematic analysis. This involved tabulating descriptions of elements of the data for each participant under the columns ‘visual only’, ‘transcript only’ and ‘visuo‐textual combined’. AM and IH then discussed the coding, ensuring critical discussion of shared and divergent ideas rather than consensus coding.

We then completed level two analysis through collaborative discussion about patterns across the visual, textual and combined data for all participants. Next, AM revisited individual participants' data to sense check the initial findings. This aligned with Brown's (2018) iterative spiral process of analysis, where the researcher gradually delves deeper into participants' experiences, as well as the flexible and organic approach of reflexive thematic analysis (Braun et al., 2019). As per reflexive thematic analysis, AM then generated themes by exploring shared meaning across the developed codes. These were reviewed and refined with IH, MT and JYFL. This iterative process within embodied inquiry draws from hermeneutics, specifically the hermeneutic circle, whereby interpretation moves between individual elements of data and the wider whole to gain a nuanced understanding of the data (Leigh & Brown, 2021) Table 1 provides an example of how meaning was constructed across the data types to generate themes.

**TABLE 1:** An example of the process of systematic visuo‐textual analysis using our data, whereby visual, textual and visuo‐textual combined data is interpreted at two levels ((1) noticing and describing, and (2) conceptualising) to generate themes (Brown & Collins, 2021).

Visual only	Textual only	Visuo‐textual combined	Conceptualisation	Theme
“Dad” and mug placed in chest area “Mum” and stars placed in chest area	Mum and dad are “the two special people to me” Cup for dad because “he loves tea” Mum “reminds me of stars”	Parents are special people, represented close to heart with mug (dad) and stars (mum)	Parents are embodied support systems (close to heart)	Knowing that your support systems are always with you
Hands gripping arms with ‘my teachers’, ‘my brother’ and ‘my other brother’ written on them Hands gripping legs with ‘my dad’ and ‘my mom’ written on them	Labelled hands “show that my supporters are still with me wherever I go”	Family and teacher represent supportive people Supportive people are always with them	Supportive people are always with me and hold me up (family and teacher)	

### Reflexivity

Reflexivity is an ongoing process (Engward & Goldspink, 2020) of self‐evaluation of one's positionality as a researcher (Mitchell et al., 2018) and how this positionality influences the lens through which we approach research. Our research team represents a range of ‘insider‐outsider’ perspectives, including Muslim and non‐Muslim researchers. The facilitators (AM, EK) and analysts (AM, IH) of body mapping are all non‐religious, white British women without children, but with experience working with children. To try and use our positions to advocate for children's voices (Caldairou‐Bessette et al., 2020), we prioritised participants' own depictions and words during analysis, giving children ‘interpretive authority’ (Carter & Ford, 2013). Systematic visuo‐textual analysis aided this, as weaving between noticing and describing (level 1) and conceptualising (level 2) meant that we checked that our interpretations aligned with participants'. We therefore came to view our findings as ‘sensitive and reflexive co‐construction’ (Carter & Ford, 2013, p. 10) between ourselves and the participants.

### Ethical considerations

Ethical approval was granted by the Queen Mary University of London Research Ethics Board (approval date: 09 October 2023; ref: QME23.0042). Parents provided written consent for their own participation and for their children. Children also provided written assent.

## RESULTS

### Sample characteristics

Nine families participated in the study (nine mothers, two fathers and 12 children: five girls, seven boys). Whilst we aimed to recruit 8–12‐year‐olds, one 7‐year‐old took part as their birthday was less than one month away at the time of data collection. The age range was therefore 7–12 (mean = 10.1, median = 11). Most families lived in Newham (*n* = 8 families) and were Pakistani (*n* = 5), Bangladeshi (*n* = 3) or Black African (*n* = 1). Of the seven families who provided this information, all described their family structure as a nuclear family, consisting of two biological parents and children. Of the seven families who specified, the number of children ranged from two to four (median = 3). Of the seven families, children were either born in the UK or moved there before age 2 (*n* = 9). Of the six families who specified, living situations included living in homes they owned (17%, *n* = 1 family) or living in private rented (50%, *n* = 3), council rented (17%, *n* = 1) or housing association (17%, *n* = 1) accommodation. From the seven families who specified, 70% of child participants received free school meals (*n* = 7/10 children). Participant demographic information is summarised in Table 2.

**TABLE 2:** Sample demographic information.

Family	Ethnicity	Borough of residence	Participant ID	Gender	Age	Length of time in UK
1	Pakistan	Newham	2B	Girl	12	Born in UK
			2C	Boy	11	N/A
2	Bangladeshi	Newham	3B	Boy	12	Born in UK
3	Pakistani	Redbridge	4A	Boy	11	Born in UK
			4B	Boy	8	Born in UK
4	Bangladeshi	Newham	5B	Girl	8	Born in UK
5	Bangladeshi	Newham	6B	Boy	12	10+ years
			6C	Boy	8	Born in UK
6	Pakistani	Newham	7B	Girl	9	N/A
7	Pakistani	Newham	9B	Girl	12	N/A
8	Black African	Newham	10B	Girl	11	Born in UK
9	Pakistani	Newham	12B	Boy	7	Born in UK

*Note*: N/A denotes that a participant did not provide this information in the demographic form.

### Themes

Findings from body mapping with children relate to two categories: ‘conceptualising resilience’ (RQ1) and ‘personal and social resources for resilience’ (RQ2). The themes within these categories are described below.

### RQ1. Conceptualising resilience

#### Acknowledging vulnerability

**FIGURE 1:**
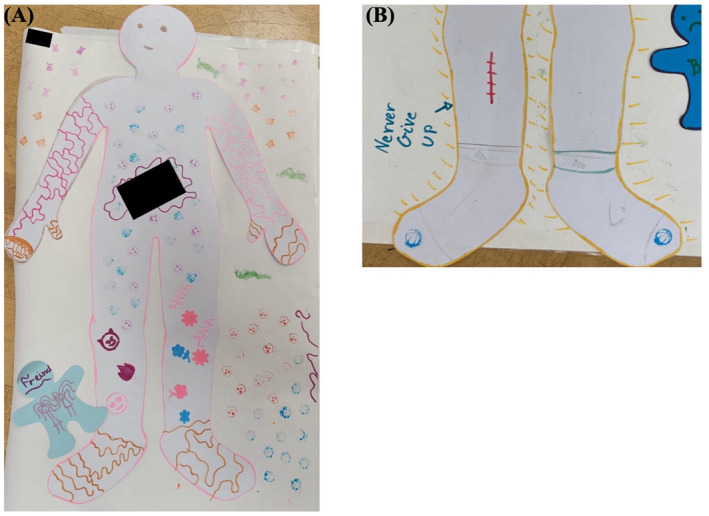
Examples of body maps from Participants 7B (A) and 3B (B) which display scars, physical injuries and nervousness.

This theme reflects depictions of resilience not as the absence of difficulty, but as the capacity to carry and deal with vulnerability. Five participants represented this through scars, physical injuries and nervousness, suggesting an understanding of resilience as something which is experienced in the context of adversity. For example, one participant ‘*drew a scar because that represents that even if you're hurt*, *you don't need to backen down*’ (*Participant* 3B, Figure 1B), reflecting resilience as overcoming challenges. One participant placed ghost and octopus stamps outside her body map to represent being ‘nervous’, contrasting them with butterfly and rabbit stamps representing ‘peace’ (Participant 7B, Figure 1A), evoking the co‐existence of these emotional experiences as part of resilience.

**FIGURE 2:**
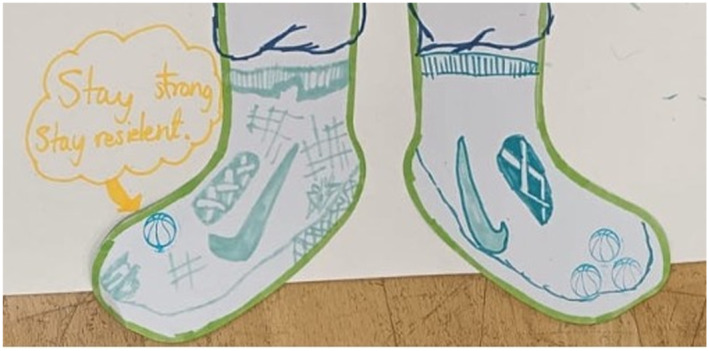
The damaged footwear of Participant 6B's body map.

Material items were also used to convey inner emotional experiences. Damaged shoes, drawn by three participants, were described as symbols of gratitude and perseverance. As one participant explained, ‘*he is still grateful and has remained resilient despite the damage on his footwear*’ (*Participant* 6C, Figure 2). These depictions resist narratives of strong and invulnerable children, instead positioning resilience as an embodied negotiation of challenges and perseverance.

#### Embodiment of nature

**FIGURE 3:**
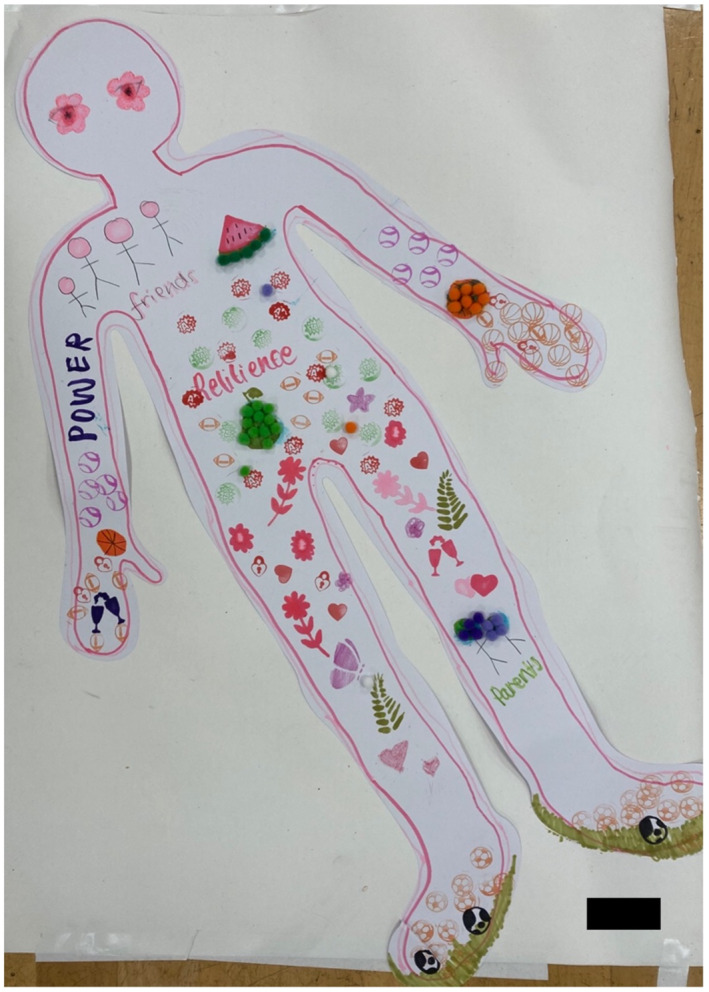
Participant 2B's body map, which contains natural imagery.

This theme captures how natural imagery was used to symbolise growth, calmness and strength, suggesting resilience as something which can be cultivated. Five body maps included symbols of nature, including flowers, plants and butterflies—evocative of peace and transformation. One child compared resilience to planting flowers, explaining that ‘*when you're resilient*, *you can*, *like*, *plant flowers because like*, *it grows with you*’ (*Participant* 2B, Figure 3), suggesting that resilience requires care and nurturing to develop over time. Animals were also used symbolically: as above, rabbits and butterflies represented peace (participant 7B). Beyond aesthetic choices, these metaphorical representations suggest how children make sense of resilience in relation to the natural world, drawing from their sensory experiences of nature as a source of wellbeing.

#### Striving to perform well in school

This theme explores how academic achievement was expressed as part of resilience, reflecting societal values of this related to self‐worth. Three participants expressed a desire to perform well in school, two of whom referenced teachers as sources of resilience. This was shown through symbols to represent teachers and academic achievement. One participant placed ‘A+’ stamps across the chest area of her body map to show ‘*grades in school*, *to be smart*’ (*Participant* 2B), this central location suggesting academic success to be integral to how she understood resilience. Similarly, one child placed ‘terrific’ stamps on their body map arms to represent how a teacher's affirmations made them feel supported: “*whenever I'm doing something at school and like an exam*, *there's someone to* *support me and then he says ‘you did well’”* (*Participant* 12B). These depictions reveal how children understood resilience in relation to their social worlds, with school being an institutional setting in which performance and praise impact children's wellbeing. This alludes to academic settings as environments in which resilience can be both compromised and supported.

#### Striving for an ideal of health and wellbeing

This theme reflects depictions of resilience through the lens of personal responsibility. Two participants, both girls, expressed how they ‘should’ behave, particularly making the ‘right’ choices, to maintain their health and wellbeing. Their body maps associated resilience with certain behaviours, including healthy eating habits and emotion regulation. One participant drew fruit ‐ a pear and a piece of watermelon ‐ in the torso and chest, respectively, explaining that ‘*the fruits show that you need to be healthy*’ (*Participant* 2B, Figure 3). This framed health as a disciplined act, and something which one should strive for to be resilient. Another child placed butterfly and rabbit stamps around their body map to show peace ‘*because you have to be calm*. *You can't be hyper*. *You just have to be peaceful*. *And I added butterflies because they don't make that noise*. *And I did rabbits because they're white*, *and peace is normally white*’ (*Participant* 7B, Figure 1A). This representation linked resilience to calmness, framing emotion regulation as a necessary response to difficult experiences.

### RQ2: Personal and social resources for resilience

#### Embodied strength and positive self‐talk

**FIGURE 4:**
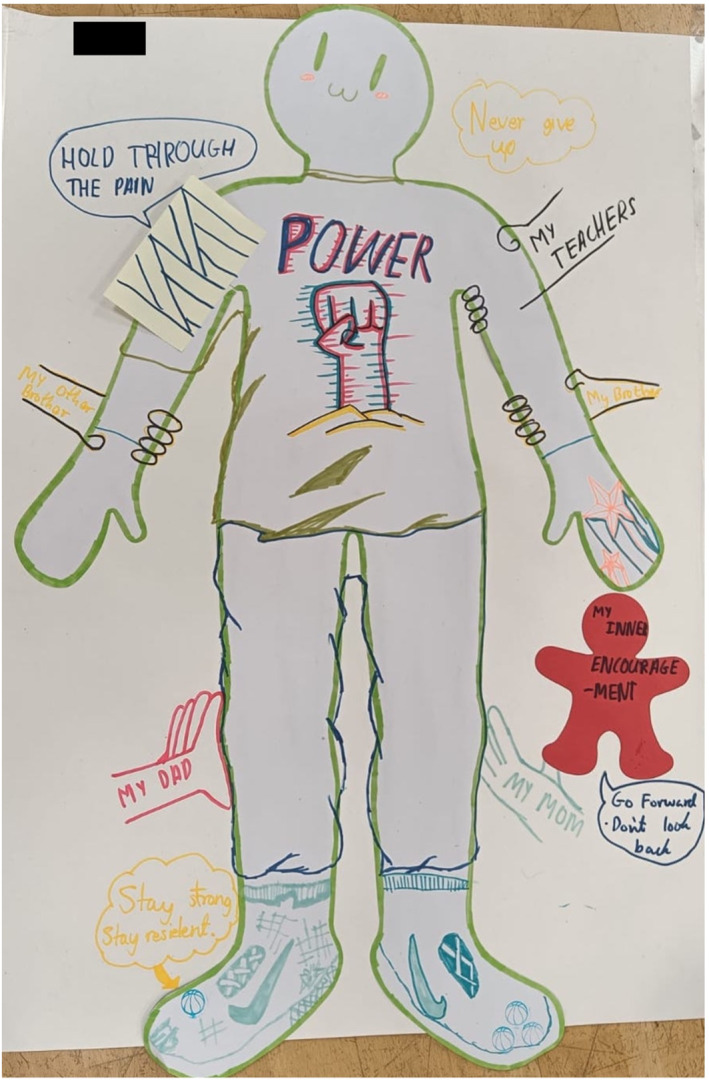
Participant 6B's body map, with ‘my inner encouragement’ depicted as an external figure on the left‐hand side of the map.

**FIGURE 5:**
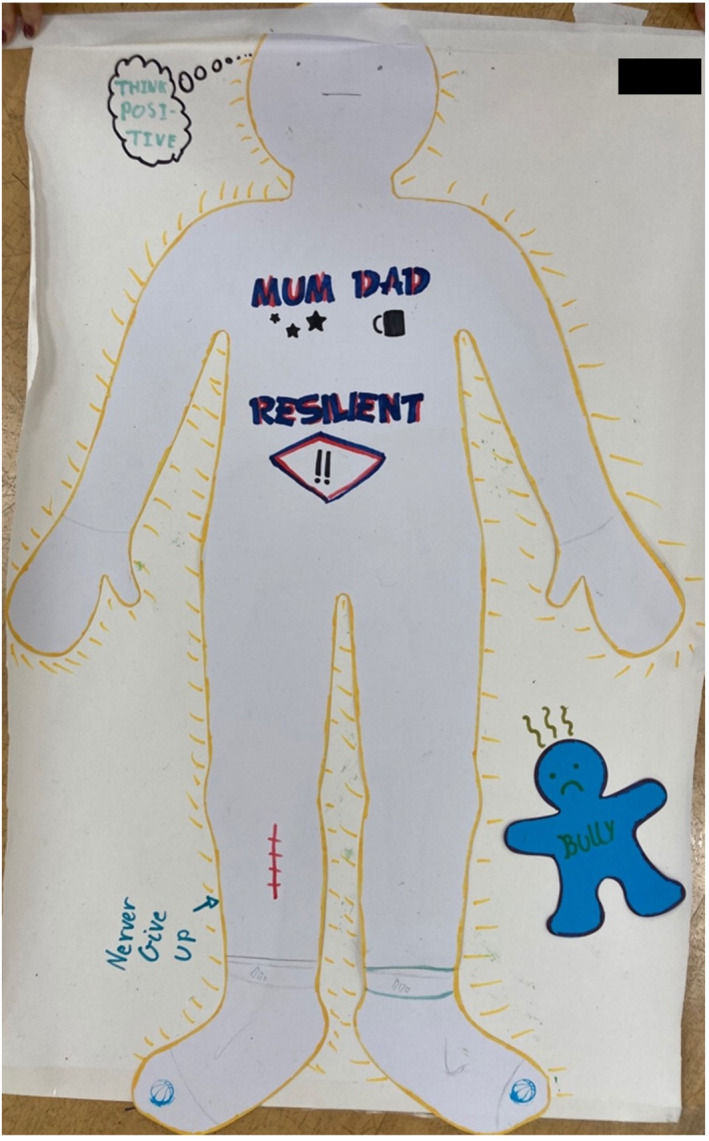
Participant 3B's body map, with parents depicted as embodied sources of resilience.

This theme relates to children's representations of resilience internal strength, held within the body and supported through internal dialogue. For some participants, the body was used as a symbol of emotional endurance. One child wrote ‘hold through the pain’ next to a bandaged arm on their body map (*Participant* 6B, Figure 4), symbolising the emotional strength needed to overcome physical pain. Another child ‘*drew a superhero on my body map*, *showing how strong and brave I am*, *like when I face tough things*’ (*Participant* 4C), likening resilience to cultural metaphors of heroism. Others visualised resilience in response to external threats, such as personal triumph over bullying; one child ‘*drew a bully and then I drew these spark things and showing that technically I'm superior to a bully because bullies just make people sadder*, *but I'm resilient*’ (*Participant* 3B, Figure 5).

Positive self‐talk was also depicted as an internal resource by several children, including using the body maps to remind themselves of their own resilience. This capacity to overcome difficulties was conceptualised as ‘my inner encouragement’ by one participant who depicted this as an external figure next to his body map. This child also depicted various people holding onto his body map, reflecting the need for both intrapersonal and interpersonal resources: ‘*there's people supporting me and there's also my own feelings*’ (*Participant* 6B, Figure 4). These representations suggest that whilst children drew from dominant discourses of individual strength, they also viewed supportive relationships as an aspect of resilience.

#### Role models and learning through sport

This theme relates to literal and symbolic representations of sport as a source of enjoyment and resilience, through which resilience is learnt and enacted. Some participants literally mapped sport onto their body maps to show how different body parts are used. One participant placed basketball stamps on the hands, explaining that ‘*I like basketball as well and you need your hands when you play it*’ (*Participant* 2B, Figure 3).

**FIGURE 6:**
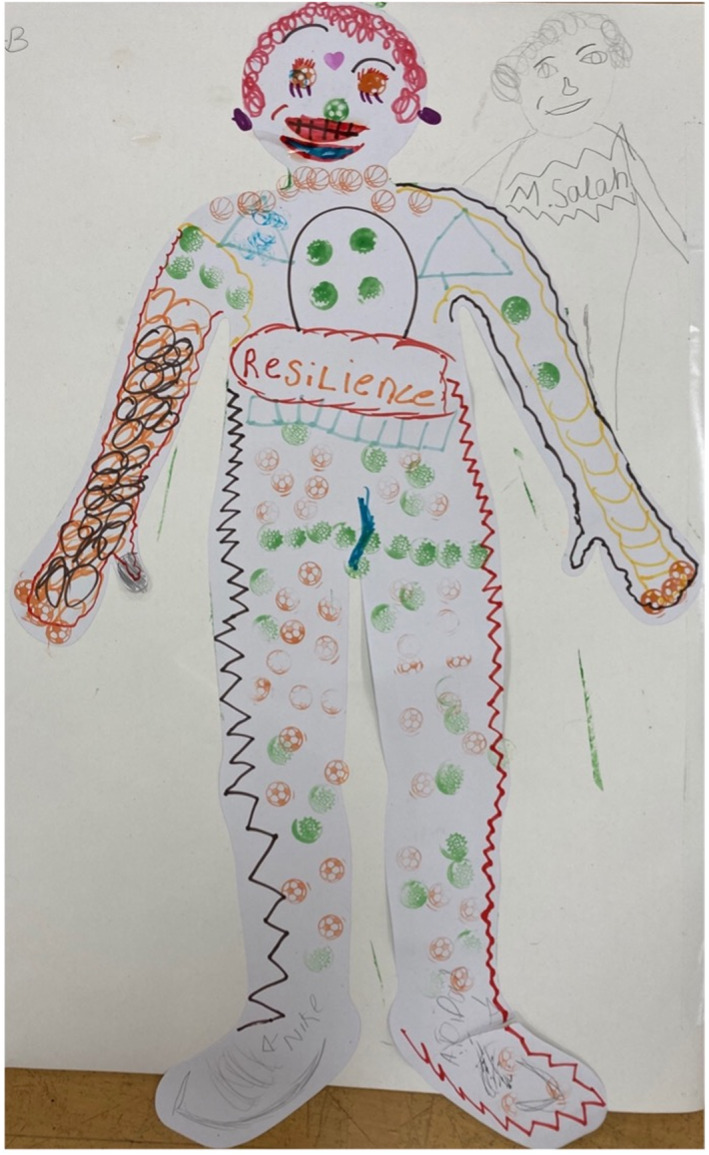
Participant 4B's body map, with footballer Mohammad Salah depicted standing over the left shoulder.

Sport was also used for participants to reflect on resilience through figures they admire. For example, one participant depicted a famous footballer as a figure watching over their body map, representing him as a role model and as symbolic of a capacity to persevere: ‘*And the person next to me*, *that's Mohammed Salah*. *He's one of my favourite footballers*. *He doesn't necessarily* *support me because he probably doesn't even know I exist*. *But he's like an inspiration to me*. *And I notice that whenever he falls down*, *he just gets back up again*. *He doesn't start crying on the floor*’ (*Participant* 4B, Figure 6).

This metaphor of perseverance was reinforced through football stamps across the legs and torso of this participant's body map: ‘*The reason why there's so many footballs on it is because in football*, *no matter how many times you fall down*, *you still have to get back up again and you can't just stay on the floor*’ (*Participant* 4B, Figure 6). Here, interestingly despite being a team sport, football was constructed as a symbolic representation of resilience as individual perseverance, and as an activity through resilience can be personally developed. Combined, these narratives suggest an understanding of resilience as developed through one's own actions, as well as being shaped by perceived role models.

#### Knowing that your support systems are always with you

This theme captures how children expressed resilience as connected to supportive figures, including family, friends and teachers. Children often depicted these support systems close to the heart or integrated into their body maps, suggesting the centrality of these relationships. One participant wrote ‘mum’ and ‘dad’ across the chest and explained that ‘*the two special people to me are my dad and my mum*. *The reason I drew a cup for my dad is because he loves tea and for my mum because she reminds me of stars*’ (*Participant* 3B, Figure 5). Teachers were depicted as sources of emotional and academic support by some participants. One participant used ‘A+’ stamps ‘*where the heart is*’ to represent their teacher (*Participant* 2C). Another child explained how their teachers are a source of resilience for them in the context of educational achievement: ‘*My teachers are supportive and this encourages me to be resilient and do my best in school work*’ (*Participant* 10B).

In other instances, children portrayed supportive people as physically, and metaphorically, holding them up and supporting their resilience. One participant drew arms labelled ‘my mom’, ‘my dad’, ‘my brother, ‘my other brother’ and ‘my teachers’ holding onto the arms and legs of his body map, explaining that ‘*I drew the hands on me to show that my supporters are still with me wherever I go*’ (*Participant* 6B, Figure 4). Another participant drew her mother next to her body map ‘*because she makes me resilient*’ (*Participant* 10B). This resonates with the idea that resilience is born from the internalisation of positive relations. These narratives demonstrate participants' perspectives on resilience as relational, with support systems sometimes depicted as internalised sources of strength. In addition to the representations of individual perseverance, these show children's understandings of resilience as something which can be developed and sustained through supportive relationships.

## DISCUSSION

This study demonstrates the applicability of body mapping as a research method with children, complementing traditional qualitative and quantitative methods. We found that children viewed resilience both as personal strength—relating it to nature, sports, physical strength and health—and as dependent on external support from family, friends, teachers and more distant role models. Our findings contribute to understanding resilience by: (a) by focusing on middle childhood, a developmental phase marked by both increased risk (onset of emotional and behavioural difficulties) and opportunity (greater plasticity and learning of resilience strategies) (Cohen Kadosh et al., 2013), and (b) by involving children from deprived areas who may face social adversity, yet are underrepresented in resilience research.

Children perceived vulnerability as part of resilience, often expressed through managing physical scars and injuries (RQ1). This alludes to mental health as an interrelationship between the mind, body and environment, coherent with Krieger's (1994, 2001) ecosocial theory, which suggests that we biologically incorporate our material and social worlds—that is, external experiences which occur at interpersonal, community, national and global levels across the life‐course may result in bodily changes (Krieger, 1994, 2001). Children expressed that resilience is not about being an ‘invulnerable child’ (Anthony & Cohler, 1987), but about acknowledging challenges and having the capacity and resources to navigate them. This is pertinent given that many participants lived in areas of high relative deprivation (based on Index of Multiple Deprivation data from the first half of their postcodes). Experiences of racial and cultural discrimination may also be present in their lives; police data shows that Muslims made up between 42% and 50% of religious hate crime victims in England and Wales between 2020 and 2023 (Gohir, 2024). Such threats influence parental decisions about children's activities, affecting children's social integration and community cohesion (Gohir, 2024). Policies, educational programmes and awareness‐raising initiatives are needed to address systemic and enacted violence. Educational programmes in school settings to tackle Islamophobia may be particularly beneficial both for preventing racism and supporting the mental health and wellbeing of Muslim students (Abu Khalaf et al., 2023; Saada, 2024).

Despite growing up in urban environments, children depicted imagery of nature on their body maps, consistent with previous research using body mapping with children in East London as part of the Development of Emotional Resilience study. Children depicted nature and fantasy as reminders of resilience, including animals, mythical creatures and flowers (Murray, Smith Scott, et al., 2025). The association of resilience with nature resonates with growing evidence of the therapeutic benefits of green and blue spaces (Bell et al., 2018) and nature‐based interventions for youth mental health and resilience (Moula et al., 2022; Obeng et al., 2023; Overbey et al., 2023), although more robust research is needed (Overbey et al., 2023). Research on how children from minoritised urban populations experience and access nature, and how nature‐based interventions can be designed in culturally meaningful ways, would be of benefit.

Children also linked resilience to school performance and health and wellbeing, possibly reflecting the impact of institutional and societal norms and structures (RQ1). The theme of striving to perform well in school suggests resilience as linked to a sense of achievement and validation. This aligns with Gartland et al.’s (2019) systematic review, which found that academic engagement, along with a safe school environment and positive relationships with teachers, are associated with resilient outcomes in children experiencing poverty. Children's emphasis on health and wellbeing may reflect cultural discourse on personal responsibility, self‐regulation and healthy living. Research shows that children perceive exercise and healthy food as important aspects of health (Fortuin et al., 2024; Piko & Bak, 2006; Pridmore & Bendelow, 1995) and resilience, along with other basic needs. Future research with youth from minoritised populations could examine how children from minoritised populations negotiate normative expectations related to health and education, especially within contexts of systemic disadvantage.

Children expressed personal and social resources as part of resilience (RQ2), aligning with conceptualisations of resilience in adult mental health research (Ayed et al., 2018). As a personal resource, resilience through embodied strength appeared across themes in our study, comparable with research with children and parents describing resilience as emotional fortitude (McDonald et al., 2019). As McDonald et al. (2019) note, it is important that children understand that their resilience is not a personal responsibility, but also about expressing emotions and having access to support systems and resources. As a social resource (Meyer & Mueser, 2011), children in our study commonly depicted resilience through embodied and external support systems on body maps, including relationships with caregivers and broader social support networks. Many children also expressed enjoyment of sports, particularly team sports, and used football as a metaphor for resilience. Some studies have shown associations between participation in sports and increased resilience in young people (Caldarella et al., 2019; Gürkan et al., 2024; Sheng et al., 2024). In line with a resource‐oriented approach, our findings support the need for policy and practice which prioritise investment in supportive social environments and reflect children's views on sources of resilience. This is important given that many resilience‐based interventions focus on improving individual functioning rather than addressing broader socioecological factors that impact wellbeing (Lorenc et al., 2020).

### Strengths and limitations

As a complex process with multiple dimensions, resilience can be challenging to conceptualise and articulate, especially for children who may not be able to access vocabulary that reflects complex experiences (Gorell Barnes et al., 2024; James, 2000; Perry & Medina, 2015). A strength of our study is that body mapping prioritised children's ‘ideas, beliefs, and metaphors’ (Pridmore & Bendelow, 1995, p. 473) and ‘shifted the power balance between researcher and participant’ (Dew et al., 2018, p. 18). The flexibility of our approach—offering pre‐made materials, freehand drawing and being able to draw within or outside the body map outlines—encouraged agency as participants could choose how they engaged and their distance from the subject (Gorell Barnes et al., 2024). This extended to the post‐body mapping discussion, as all questions were guided by participants' depictions rather than researchers' pre‐existing assumptions about resilience.

The limitations of this study must also be considered. First, although drawing offers a non‐verbal means of expression and can ease ‘stress associated with oral communications’ (Tumanyan & Huuki, 2020, p. 388), it can also engender self‐consciousness about one's artistic abilities (Davy et al., 2014). We made sure to remind participants that the activity was not dependent upon their artistic skills. Second, as children were sometimes shy or found it difficult to describe their body maps, in‐depth post‐body mapping interviews may have supported our findings (Vaughan et al., 2023). Third, whilst we recruited families, we focused on resilience in children. However, many of the findings from parents suggest children's resilience to be interconnected with resilience across the family system (Murray, Hensler, et al., 2025). Family resilience remains underexplored in research, and therefore future studies could triangulate our findings.

## CONCLUSION

To conclude, this study contributed arts‐based knowledge about how children define and understand resilience. Through body mapping, children described personal and relational aspects of resilience, reflecting both dominant concepts and abstract understandings of it. This study also contributed to the literature on the use of body mapping with children, and showed its value for embodied and creative engagement in mental health research.

## AUTHOR CONTRIBUTIONS

**Aisling Murray**: Conceptualization; data curation; formal analysis; investigation; methodology; project administration; visualization; writing—original draft; writing—review and editing. **Imogen I.Hensler**: Data curation; formal analysis; writing — review and editing. **Eleanor Keiller**: Investigation; methodology; writing—review and editing. **Faiza Durrani**: Investigation; methodology; supervision; writing—review and editing. **Patrisiya Ali Taleb**: Investigation; methodology; supervision; writing—review and editing. **Maria Grazia Turri**: Methodology; supervision; validation; writing—review and editing. **Jennifer Y. F. Lau**: Conceptualization; investigation; methodology; supervision; validation; writing—review and editing.

## CONFLICT OF INTEREST STATEMENT

The authors declare no conflicts of interest.

## ETHICAL CONSIDERATIONS

This study was approved by the Queen Mary Ethics of Research Committee at Queen Mary University of London (approval date: 09 October 2023; ref: QME23.0042). Written informed consent to participate in this study was provided by parents for them and their children. Children also provided written assent prior to data collection.

## Data Availability

The data that support the findings of this study are available from the corresponding author upon reasonable request.
